# Preparing a People: Climate Change and Public Health

**DOI:** 10.1289/ehp.119-a166

**Published:** 2011-04

**Authors:** Catherine M. Cooney

**Affiliations:** **Catherine M. Cooney**, a science writer based in Washington, DC, has written for *Environmental Science & Technology* and *Chemical Watch*

Almost 700 people died from heat-related stress during the catastrophic 1995 heat wave in Chicago, Illinois.^1^ The three-day weather event saw 24-hour mean average temperatures of 87.2°F; the heat reached triple digits on two days, and there was little relief at night.^2^ Many people succumbed to heart attack and dehydration, while others collapsed during severe episodes of existing respiratory conditions.^3^ The death toll in the summer of 1995 gave Chicagoans a clear picture of how a surge in hot weather can affect human health.

A decade later, Mayor Richard Daley launched an extensive program that brought together city agencies, academics, and scientists to develop a Climate Change Action Plan to help reduce the city’s contribution to climate change.^4^ Much of the plan focuses on sustainable mitigation actions such as planting trees and training workers to install renewable energy technologies. Within that plan, however, is a climate change adaptation strategy with a goal of preparing the city and its residents for future unusual weather events associated with climate change.^5^

Chicago is one of several large cities with climate action plans in place—others include New York City, San Francisco, Sydney, and Mexico City.^6^ Like Chicago’s, these plans promote mitigation and sustainability. Much of the adaptation portion of these initiatives is aimed at the built environment—buildings, highways, and facilities. But officials in these cities are beginning to talk about the public health cobenefits from their action plans, and public health advocates are speaking up and pushing for programs designed to prepare for or prevent climate-sensitive disease and illness.

## A Need to Plan

Changes in climate patterns that have been seen in the United States and around the world are likely to be connected with sweltering heat waves in Chicago, Milwaukee, and other locales, many scientists agree.^7^,^8^,^9^ Yet disease and mortality tied with heat are not the only public health dangers arising from climate change. Researchers have noted a clear link between increased rainfall and a rise in diarrheal illnesses following heavy rains that overwhelm sewers and water systems.^7^ Warmer year-round temperatures—milder winters, earlier spring thaws, and later frosts—have lengthened pollen seasons^10^ and brought forth a surge in toxic plants such as poison ivy.^11^ In the southeast United States, warming waters are expected to encourage the growth of the aquatic bacterium *Vibrio vulnificus*, which can cause infections potentially leading to a loss of limbs and possibly death if not treated.^12^ Changes in the reach of tropical diseases such as malaria, dengue, West Nile virus, schistosomiasis, leprosy, and cholera also are predicted, as vectors and ecosystems adapt to warmer climates.^7^ And changes in the composition and interaction of air pollutants such as ozone, particulate matter, and aeroallergens are expected to heighten human health effects of these pollutants.^13^

“These are not distant lands and faraway times; they are very much immediate issues, from worsening air pollution to infectious diseases to increases in heat events and extreme rainfall events. All of these [changes] affect human health outcomes, and all are expected to increase . . . as climate change continues,” said Kim Knowlton, senior scientist with the Health and Environment program of the Natural Resources Defense Council, during a spring 2010 webinar. The webinar, “Climate Change: Mastering the Public Health Role” was cosponsored by the American Public Health Association (APHA), the National Association of County and City Health Officials (NACCHO), the Association of State and Territorial Health Officials, and the Society for Public Health Education.^14^

Chicago’s efforts notwithstanding, adaptation planning is still very much a new idea for U.S. cities.^6^,^15^ The rise in illness and mortality reported during heat waves, hurricanes, and other extreme weather events indicates the public health community in the United States, and elsewhere, is not yet prepared to handle the increase in human disease predicted to arise with climate change, according to many of the public health scientists interviewed for this story. “Public health organizations are not very well prepared, which is reflected in the numbers of injuries, illnesses, and deaths due to diseases sensitive to weather and climate,” said Kristie L. Ebi, an independent consultant, speaking during the APHA webinar.

Public health groups and some researchers are starting to speak out about this lack of preparation. In fall 2007, upon the launch of an APHA initiative to develop key recommendations for climate change adaptation, association executive director Georges C. Benjamin said, “Climate change is one of the most serious public health threats facing our nation. Yet few Americans are aware of the very real consequences of climate change on the health of our communities, our families, and our children.”^16^

The following summer, Edward Maibach, director of the Center for Climate Change Communication at George Mason University, and colleagues published a report of their survey of 133 directors of local public health departments on departmental climate change adaptation activities and attitudes. They wrote, “[O]ur survey points to relatively widespread awareness of the importance of climate change for public health among directors of local health departments, but far lower levels of actual preparedness or planned activities to detect, prevent and ameliorate climate-associated health problems.”^17^ The authors pointed to lack of knowledge about climate change, more pressing immediate priorities, and a chronic lack of resources as potential factors undermining departments’ ability to plan for climate change.

Cecil Wilson, president of the American Medical Association, and coauthor Paul R. Epstein, associate director of the Center for Health and the Global Environment at Harvard Medical School, followed up with comments in a December 2010 article for the *Huffington Post*, noting that “climate change is hazardous to our health.” The American Medical Association is working actively to educate health care professionals about the projected rise in climate-related illness, Wilson and Epstein added.^18^

“Understanding the health impacts of climate change is one of the most vital pieces of information we need to make sound decisions about climate change adaptation,” says Michelle Bell, an associate professor of environmental health at Yale University. “We hear about sea-level rise, changes in vegetation, forest fires . . . but compared with other impacts, we hear very little about human health. I think the general public and decision makers would very much like to hear about the health implications.”

## What Is Adaptation?

Adaptation refers to actions that countries, communities, organizations, and individuals can take to prepare themselves for and protect themselves against the impacts of climate change. Because health impacts vary widely depending on geographic location, planning for climate-related health consequences should occur on local to national levels.^17^

A few cities—primarily either those that have recently experienced devastating weather-related events or those located along the coasts—are preparing climate change adaptation plans, but still fewer of these include actions the public health community should be taking. Yet the public health viewpoint is vital to the success of adaptation plans, notes George Luber, associate director for climate change at the U.S. Centers for Disease Control and Prevention (CDC), who moderated the APHA webinar. “Some adaptation working groups aren’t making the connection with public health,” Luber said at the webinar, even though “health is so critical to measure the effects of the plans.”

JoAnn Carmin, an associate professor of environmental policy and planning at the Massachusetts Institute of Technology, has studied adaptation programs under way in U.S. cities. Although few cities have a formal adaptation program, “it depends on what we call ‘adaptation,’” Carmin says. “If we started talking about ‘healthy cities’ programs, ‘green cities’ programs, ‘sustainable cities’ programs, things start to change a little.” Air pollution reduction strategies, “cooling stations” (e.g., air-conditioned meeting rooms in apartment complexes or traveling buses), or more informed emergency responders—programs included in some state- and city-level climate change action plans—also fall under the rubric of adaptation, Carmin says.

Those cities that have developed adaptation plans have been openly experimenting to figure out what works, according to Carmin. Chicago, for example, “saw an issue that was really important to the residents,” Carmin says. “They were very methodical about the way they went about [developing their plan], and it is being done in a way that lets other cities find out how they did it.”

Released in fall 2008, Chicago’s Climate Action Plan^4^ is made up of five strategies: adaptation, energy-efficient buildings, transportation options, renewable energy, and waste management. The city relied on three separate analyses to support the plan: a comparison of potential climate change scenarios in Chicago under higher and lower greenhouse gas emissions, an assessment of Chicago’s economic risk under both emissions scenarios, and a prioritization of potential impacts based on the likelihood and local consequences of occurrence.^19^

Although much of Chicago’s plan details actions to improve the sustainability of the built infrastructure—buildings, housing, and transportation—the public health component was very much part of the planning process, says Cortland Lohff, medical director for environmental health with the Chicago Department of Public Health. Many adaptation actions designed to lower the impact of the city’s heat island effect,^20^ such as opening new cooling centers and issuing heat advisories that encourage people to stay hydrated and cool, also can improve public health conditions by minimizing the physical effects of extreme heat exposure.

Officials in New York, Boston, and Portland also are drafting climate change adaptation initiatives, Carmin says. The city of Keene, New Hampshire, was one of the first smaller communities to draft an adaptation plan. Keene city officials worked with ICLEI—Local Governments for Sustainability,^21^ an international association of more than 1,200 local government members committed to sustainable development, on a pilot demonstration under ICLEI’s Climate Resilient Communities Program. This program lays out five steps to get cities moving toward an adaptation plan: conduct a climate resiliency study, set preparedness goals, develop an actual climate preparedness plan, publish and implement the plan, and monitor and reevaluate resiliency.

“As adaptation moves forward in the discussions, you will begin to see that cities don’t need to reinvent the wheel. They will need to use the programs and initiatives they are using now, but will need to look at those actions with a different lens,” Carmin says.

## Working Together

In northern California, officials with Alameda County’s Public Health Department are coordinating with agencies in several nearby counties—health departments and other county agencies, including those responsible for transportation—on small steps toward climate change mitigation as part of ongoing sustainability efforts, says Sandra Witt, deputy director of the Alameda County Public Health Department. The department has been active in trying to incorporate health and socioeconomic equity into local climate action plans.

A large part of the county’s sustainability and climate change work targets poor people of color living in areas close to the freeways, the Oakland seaport, and industrial facilities. State data show that these communities are exposed to much higher levels of air pollution and are already suffering with more cases of asthma, respiratory illness, and cancer, Witt says.^22^ “From a public health perspective, we are really concerned about the health impacts for everyone and realizing that the low-income and people of color will be impacted the most,” Witt says.

These northern California counties, like most in the country, are working without climate change funding support from the state or from the federal government, Witt adds. “We are trying to put climate change adaptation into work that we are [already] doing, because a lot of the sustainability programs will also help reduce illnesses exacerbated by climate change,” she says. “It is a matter of doing what you can do within the confines you have.”

Witt says Alameda County is just starting to partner with the Adapting to Rising Tides planning effort to conduct a vulnerability assessment on certain likely scenarios and determine the response of the Public Health Department. The Adapting to Rising Tides program is a partnership of the San Francisco Bay Conservation and Development Commission and the National Oceanic and Atmospheric Administration (NOAA) Coastal Services Center that is designed to help Bay Area communities begin planning for sea-level rise.

Alameda is one of many counties collaborating with NACCHO on modeling and other tools to help build adaptation programs. NACCHO recently selected six other county health departments located throughout the United States to conduct one-year demonstration projects. The goal of the projects is to illustrate steps that address a basic challenge of adaptation measures: a lack of coordination and communication between disparate agencies and programs that are all involved in addressing the public health consequences of climate change.^23^

Ebi, who has also identified adaptation options for states and for groups in low-income countries, urges scientists and practitioners to remember that public health–oriented adaptation programs must be flexible. They should be evaluated for their ability to function in a changing climate, Ebi says. “Ideally, programs make sense no matter how the climate changes,” she explains. “The challenge will be to develop actions for a climate that will differ from what is currently considered normal.”

## Branching Out

Patrick Kinney, a professor of environmental health sciences at Columbia University’s Mailman School of Public Health, is a bit ahead of the pack with his research focus on the changing impacts of air pollution and sensitivities to allergies and other respiratory diseases as a result of climate change. He is a coauthor of one of the first papers to examine ozone-related health impacts that could occur with future warming in metropolitan areas.^24^

Studies on heat waves and warmer winters have dominated the research to date on climate change–related health effects, Kinney says. Beyond heat stress, researchers have not spent much time examining other impacts, leading to a situation in which there is very little knowledge that can be used to help craft adaptation plans, he says.

“I wouldn’t say we have no knowledge,” Kinney says. “We have maybe five to ten percent of what would be needed . . . to make well-informed decisions.” New studies that employ models and incorporate historical data “can help paint a picture of where human health will be compromised the most,” he adds.

In a 2009 paper, Kinney, Ebi, and coauthors published a “first pass” estimate that the United States will need to spend more than $200 million annually to address the health problems associated with climate change.^25^ Ebi says the money could be used to build on the research that has been collected so far on health effects that will be exacerbated by climate change, such as health effects from increased air pollution or increased allergens. The money is also needed to help point out how researchers can use that existing body of research to say how much ozone levels will change and how that will affect public health in a changing climate. Extramural federal funding for climate change and health research in 2009 was estimated at around $6 million per year.^25^

The Obama administration is enthusiastically pursuing a new national adaptation policy that it hopes will provide public health officials with the tools they need to predict and respond to public health crises caused by rising temperatures, increased drought, more extensive rains, sea-level rise, and more days with poor air quality.^26^ The government’s mandate to develop a national policy on adaptation comes from the top, according to John Holdren, director of the White House Office of Science and Technology Policy. Speaking in December 2010 at the American Geophysical Union (AGU) annual meeting in San Francisco, Holdren noted that the White House Council on Environmental Quality had just released its *Progress Report of the Interagency Climate Change Adaptation Task Force: Recommended Actions in Support of a National Climate Change Adaptation Strategy*.^27^ The report is the first issued by the U.S. government and to some extent validates the adaptation work that county and city health departments have begun.

Jane Lubchenco, director of NOAA, agreed at the AGU meeting that adaptation was a priority for the White House. “While most of the public dialogue has been about mitigation, most of the private government dialogue has been about mitigation and adaptation, and appropriately so,” Lubchenco said.

Although adaptation planning can be most successful at the local level, a national policy can help, Carmin says. She explains, “If someone from a federal agency stands up and says, ‘This is what we do in the U.S.,’ it will make the job of people in the cities a lot easier. The government’s policy starts to legitimize what the people on the local level are doing and what they know.”

## Solid Steps

Juli Trtanj, program director of NOAA’s Ocean and Human Health Initiative, sits on the U.S. Global Change Research Program’s (USGCRP) Working Group on Climate Change and Human Health. The USGCRP, which coordinates and integrates federal research on changes in the global environment and their implications for society, is developing a new strategic plan for better incorporating and responding to society’s needs related to climate change. This new approach is in response to multiple reports issued over the past decade by the National Research Council, including the *America’s Climate Choices* series issued last fall.^28^

The working group is led by the National Institutes of Health, the CDC, and NOAA with a liaison from the White House Office of Science and Technology Policy. Trtanj says one goal of the working group is “to identify [the public health community’s] needs in terms of predictive capacity . . . such as tools for early warning systems so they can better respond way ahead of the curve instead of being caught by surprise.”

In 2007–2009 the working group held a series of “listening sessions” with public health groups and found that most groups report being interested in climate data at more regionally or locally relevant temporal and spatial scales—what’s known as “downscaled climate information.” These groups also need help managing the health surveillance data that have already been collected by state and local agencies. “To develop a policy, [cities, counties, and states] need to integrate the health surveillance data—for example, heat wave mortality or foodborne and vectorborne disease data—with climate change data,” Trtanj says.

As a first step in developing a Monitoring, Early Warning, Data Integration, and Surveillance System, the working group is collecting information on the climate-related environmental and health surveillance data that are available at the federal level. Once collected, descriptions of the data and information on where to find them will be placed on a public website. The group also plans to conduct outreach to help the medical and public health communities communicate more effectively about the risks and changes associated with climate change.

Trtanj says these adaptation tools are being developed with little or no additional funding. She says that finding funds to support climate change adaptation work is an uphill battle for several reasons, including the fact that many agencies are each doing a piece of the climate change work. “We are doing an awful lot, honestly, with blood sweat and tears and with the people who really care about this, but it is not sustainable,” Trtanj says. “Unless there is real support for this . . . it will stay a bit ad hoc, and we won’t have any comprehensive plans for understanding the human health impacts of climate change and how we will adapt to them.”

Because California is struggling with budget troubles, much of the public health–related adaptation work is done around issues that *are* being funded, Witt says. For example, climate change adaptation can become a cobenefit of programs designed to combat obesity that encourage people to get out of their cars and walk. “We want to be able to do the analysis that we need to do so we can present this issue to decision makers, but we don’t have funding for it. We are still doing it, but it is not easy,” Witt says.

One federal agency does have some money that can be used to prepare for climate change health effects. The CDC recently announced that 10 states and cities have been chosen to participate in its Climate-Ready States and Cities Initiative. The CDC will help states and cities partner with local and national climate scientists to understand potential climate change scenarios in their areas. It also will assist states and cities in developing and using models to predict local-level health impacts, to monitor health effects, and to identify the most vulnerable residents of each area. The agency is providing a total of $5.25 million to these 10 health departments over a three-year period.^29^

Elsewhere in the world, Ebi says all developing countries have done at least a limited adaptation assessment, and quite a few developed countries have national adaptation plans. These efforts should be further bolstered by an agreement reached in December 2010 at the United Nations Climate Change Conference in Cancun, Mexico, where delegates reached an accord that emphasizes the pressing need for adaptation policies.

The negotiators adopted the Cancun Adaptation Framework, which establishes an adaptation committee and a work program to address unavoidable climate impacts in vulnerable countries.^30^ The framework highlights the elements countries should be considering in adaptation action, including those focused on public health. Perhaps to highlight their commitment to the framework, Mexican officials unveiled their country’s adaptation strategy at the Cancun meeting.^31^

Examples of city-level climate change adaptation strategies also are emerging in the European Union. Like their counterparts in the United States, adaptation plans in Europe are often part of wider climate change and sustainability strategies that encompass—and in some cases are largely focused on—climate change mitigation. Madrid and Manchester have this type of integrated climate change plan, whereas London, Copenhagen, and Rotterdam have already created stand-alone adaptation strategies that exclusively target impacts of climate change.^32^

## A New Way to Frame Climate Change

In addition to money woes, a lack of data, and limited research, many local officials are challenged because their constituents don’t believe climate change is a real problem.^33^ In a draft paper on public health and communication not yet submitted for publication, Maibach and colleagues describe how a new public health “frame” for climate change—i.e., making the case that climate change is a major threat to people’s health and well-being—has potential to engage a much broader cross-section of the American public than has previously been interested in the issue. This public health frame can also help connect the complex and poorly understood topic of climate change to risks that the public already understands and accepts as important, such as asthma, vulnerability to extreme heat, foodborne illness, and infectious disease, Maibach says.

Maibach and colleagues call on public health professionals to take some responsibility about discussing the impact of climate change on health as well as climate-sensitive illnesses. Says Maibach, “By explaining that climate change is a human health threat—not just a threat to plants, penguins, and polar bears—public health professionals have a unique opportunity to enhance public engagement in the issue.”

## Figures and Tables

**Figure:**
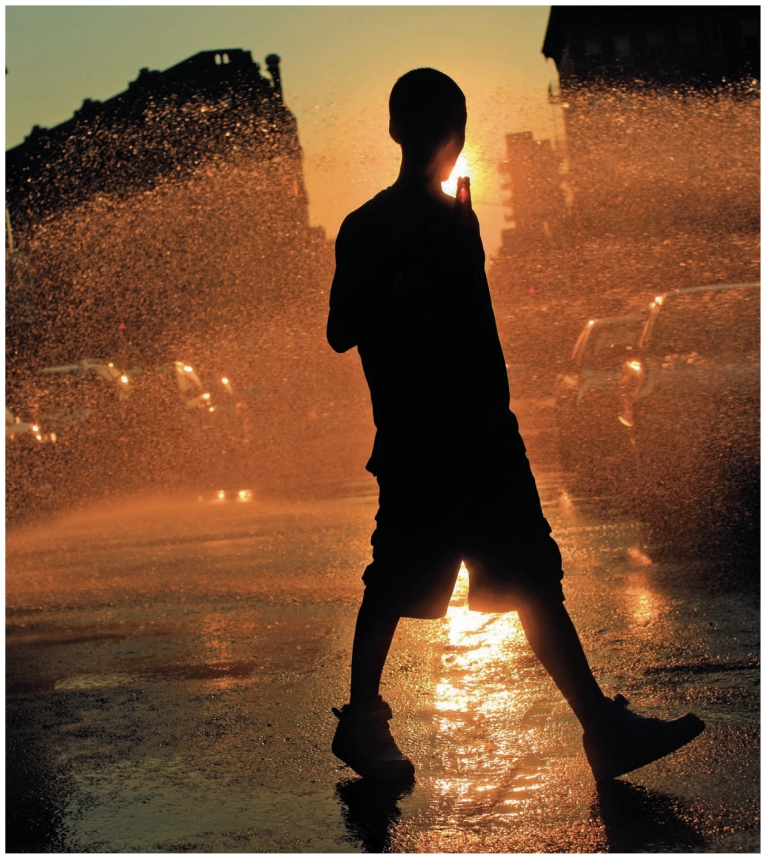
Water sprays from an open fire hydrant in Brooklyn, New York, in the midst of a July 2010 heat wave that affected much of the eastern United States. In 2007 the New York City Department of Environmental Protection first teamed up with Alianza Dominicana, a Washington Heights community organization, to educate city residents about the appropriate use of fire hydrants and other ways to stay cool during heat waves. Hydrants can be opened legally and safely if equipped with an approved spray cap, available at local firehouses.

**Figure:**
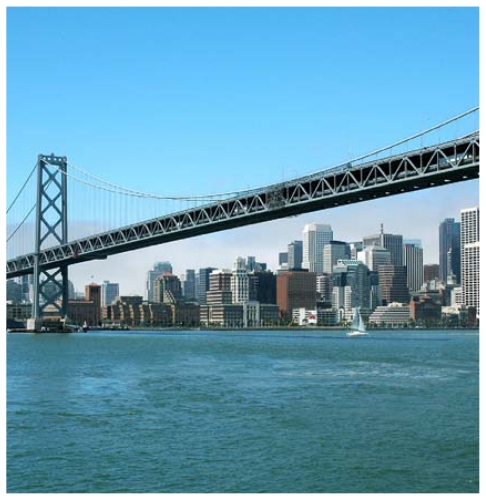
Understanding the health impacts of climate change is one of the most vital pieces of information we need to make sound decisions about climate change adaptation. We hear about sea-level rise, changes in vegetation, forest fires . . . but compared with other impacts, we hear very little about human health.” —Michelle Bell Yale University

**Figure:**
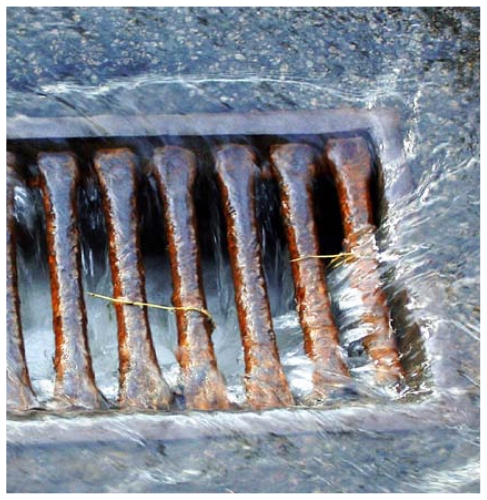
We are trying to put climate change adaptation into work that we are [already] doing, because a lot of the sustainability programs will also help reduce illnesses exacerbated by climate change. It is a matter of doing what you can do within the confines you have. —Sandra Witt Alameda County (California) Public Health Department
